# Comparative genomics to examine the endophytic potential of *Pantoea agglomerans* DAPP-PG 734

**DOI:** 10.1186/s12864-022-08966-y

**Published:** 2022-11-08

**Authors:** Arburon Sulja, Joël F. Pothier, Jochen Blom, Chiaraluce Moretti, Roberto Buonaurio, Fabio Rezzonico, Theo H. M. Smits

**Affiliations:** 1 Environmental Genomics and Systems Biology Research Group, Institute of Natural Resource Sciences (IUNR), Zurich University of Applied Sciences (ZHAW), Wädenswil, Switzerland; 2 Bioinformatics and Systems Biology, Justus-Liebig-University Giessen, Giessen, Germany; 3 Dipartimento Di Scienze Agrarie, Alimentari E Ambientali, Università Degli Studi Di Perugia, Perugia, Italy

**Keywords:** Genome, Biocontrol, Olive knot disease, Antibiotics biosynthesis, Integrative conjugative element, ICE, Secretion systems

## Abstract

*Pantoea agglomerans* DAPP-PG 734 was isolated as endophyte from knots (tumors) caused by *Pseudomonas savastanoi* pv. savastanoi DAPP-PG 722 in olive trees. To understand the plant pathogen-endophyte interaction on a genomic level, the whole genome of *P. agglomerans* DAPP-PG 734 was sequenced and annotated. The complete genome had a total size of 5′396′424 bp, containing one circular chromosome and four large circular plasmids. The aim of this study was to identify genomic features that could play a potential role in the interaction between *P. agglomerans* DAPP-PG 734 and *P. savastanoi* pv. savastanoi DAPP-PG 722. For this purpose, a comparative genomic analysis between the genome of *P. agglomerans* DAPP-PG 734 and those of related *Pantoea* spp. was carried out. In *P. agglomerans* DAPP-PG 734, gene clusters for the synthesis of the Hrp-1 type III secretion system (T3SS), type VI secretion systems (T6SS) and autoinducer, which could play an important role in a plant-pathogenic community enhancing knot formation in olive trees, were identified. Additional gene clusters for the biosynthesis of two different antibiotics, namely dapdiamide E and antibiotic B025670, which were found in regions between integrative conjugative elements (ICE), were observed. The in-depth analysis of the whole genome suggested a characterization of the *P. agglomerans* DAPP-PG 734 isolate as endophytic bacterium with biocontrol activity rather than as a plant pathogen.

**Supplementary Information:**

The online version contains supplementary material available at 10.1186/s12864-022-08966-y.

## Introduction

*Pantoea agglomerans*, a member of the family of *Erwiniaceae* [1], is one of the most commonly isolated and studied species of the genus *Pantoea*. *P. agglomerans* strains morphologically appear as straight rod-shaped Gram-negative bacteria usually producing a yellow pigment [2]. Furthermore, *P. agglomerans* cells are facultatively anaerobic, oxidase negative, they use D-glucuronate and D-tartrate as sole carbon sources and perform an alkaline reaction in malonate broth [3]. This species is widely distributed in nature and has been isolated from numerous ecological niches, primary from plant surfaces but also from flowers, seeds, vegetables, water, soil and foods [3–5]. Some strains were isolated from blood, urine, wounds and the intestine of humans and animals, like the type strain *P. agglomerans* DSM 3493^ T^ (NCTC 9381^ T^, ATCC 27155^ T^, LMG 1286^ T^), which was isolated from a knee wound in Zimbabwe [6]. Based on clinical reports as opportunistic human pathogen causing infections [7–9], all *P. agglomerans* isolates were classified as a biosafety 2 (BL-2) microorganism in Europe [10].

*P. agglomerans* was associated primarily with plants as an epiphyte or endophyte [11] but was also identified as a plant pathogen [5, 12, 13]. *P. agglomerans* pv. gypsophilae 824–1 and *P. agglomerans* pv. betae 4188 were identified as tumorigenic as they are able to provoke gall formation on gypsophila plants (*Gypsophila paniculata*) and on beet and as well on gypsophila plants, respectively [12, 13]. In *P. agglomerans* pv. gypsophilae 4188 the gall formation depends on the type III secretion system (T3SS) [14, 15], the phytohormones indole-3-acetic acid (IAA) and cytokinins [13, 16, 17], and the quorum-sensing (QS) communication system [5, 18, 19]. It was demonstrated that the olive knot endophytic bacterium *P. agglomerans* DAPP-PG 734 is able to communicate with *Pseudomonas savastanoi* pv. savastanoi DAPP-PG 722 through a QS system mediated by N-acyl-homoserine lactones (AHLs) [20], it produces a relatively high amount of IAA in vitro, while a functional Hrp-1 T3SS of *P. agglomerans* DAPP-PG 734 is required for full virulence of *P. savastanoi* pv. savastanoi DAPP-PG 722 [21, 22]. The presence of *P. savastanoi* pv. savastanoi DAPP-PG 722 strongly increases the growth *in planta* of *P. agglomerans* [20]. In contrast, the growth of *P. savastanoi* pv. savastanoi ITM105 and ITM317 was inhibited by a dominant population of *P. agglomerans* strains SC1, FL1, or MM2 probably due to the competition for space and nutrients between the two bacteria and to the antibiotic production by *P. agglomerans* [23].

Some *P. agglomerans* strains can be used in biotechnology field due to their biochemical activity against other microorganisms and their adaptability to different environmental conditions. It was shown that some *P. agglomerans* strains can produce effective antibiotics [24, 25] and thus can be used as biocontrol agent against *Erwinia amylovora*, the causal agent of fire blight of pomaceous trees [26, 27]. To date, *P. agglomerans* E325, branded as BloomTime Biological™, *P. agglomerans* P10c, registered as BlossomBless™, and *Pantoea vagans* C9-1, marketed as BlightBan C9-1™, have been successfully registered in Canada, New Zealand and in the USA as biocontrol agent against *E. amylovora* [28–30]. However, even though many different *Pantoea* spp. can produce potent antibiotics against pathogenic bacteria, the genes involved in their biosynthesis have not been completely identified and/or analyzed. Further investigation or even discovery of novel antibiotic biosynthesis genes or gene clusters may yield potentially new antimicrobial metabolites against pathogenic bacteria [31].

In 2014, several *P. agglomerans* strains, which induced a hypersensitive reaction in tobacco plants, were isolated from olive knots [21, 32]. In the present study, the complete genome assembly and annotation of *P. agglomerans* DAPP-PG 734 is reported, and the comparison of its genome with those of other *P. agglomerans* strains and related *Pantoea* spp. is performed to understand the role of *P. agglomerans* DAPP-PG 734 as an endophyte, or as a potential pathogen or as a biocontrol agent in the olive knots. Furthermore, based on the comparative genomics results, some potential antimicrobial metabolites produced by *P. agglomerans* DAPP-PG 734 are identified and their ecological role is investigated.

## Results and discussion

### General features of the *P. agglomerans* DAPP-PG 734 genome

**Fig. 1:**
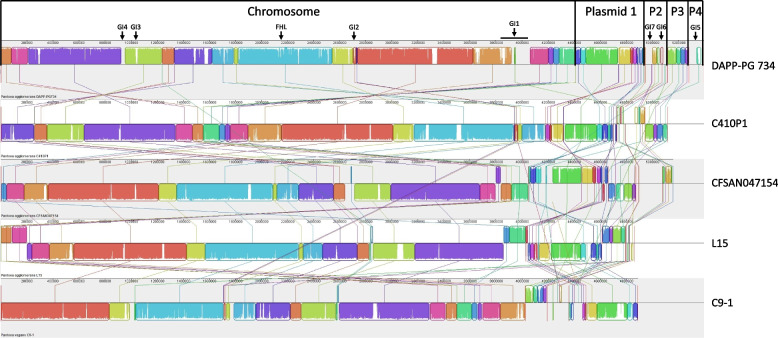
Genome alignments showing local collinear blocks among *Pantoea agglomerans* strains obtained using progressive MAUVE. *P. agglomerans* DAPP-PG 734 was compared with other closely related strains whose genomes are completely sequenced, namely *P. agglomerans* C410P1, *P. agglomerans* CFSAN047154, *P. agglomerans* L15 and *P. vagans* C9-1. Each colored block represents a locally collinear block or homologous region shared among genomes. GI: genomic island; FHL: formate hydrogenlysase; Px: Plasmid x

The assembly performed by Moretti et al. [21], only based on Illumina reads, contained 195 contigs (*N*_*50*_ = 53′927 bp) with a total sequence length of 5′365′929 nucleotides (Table 1). In this study, in order to complete the genome sequence, the genome was resequenced using the MinION sequencing. After a first assembly with Unicycler [33], eight contigs with a total genome size of 5′396′422 bases were obtained. Further manual assembly reduced the number of contigs to five circular contigs (*N*_*50*_ = 4′410′564 bp) representing the chromosome and four large plasmids with a total genome size of 5′396′424 bases. The Prokka annotation [34] resulted in a total of 76 tRNA and 22 rRNA, and 4′991 CDS. A comparison between the complete genome of *P. agglomerans* DAPP-PG 734 and those of other *P. agglomerans* strains shows that in the chromosome, large collinear blocks in a largely conserved order are present, while plasmids show a high variability (Fig. 1).

**Table 1:** Genome assembly metrics for the assembly of *Pantoea agglomerans* DAPP-PG 734 genome. The Edena assembly was reported before [21]

Assembly name	ASM71021v1	ASM71021v2
Sequencing technology	Illumina MiSeq	Illumina MiSeq + Oxford Nanopore MinION
Assembler	Edena v.3 dev. 120,926	Unicycler v.4.4.8
Coverage	37 ×	141 ×
Total sequence length (bp)	5′365′929	5′396′424
Number of contigs	195	5
*N*_*50*_	53′927	4′410′564
G + C content (%)	54.6	54.7
Annotation pipeline	-	Bakta v.1.2.4
Number of		
CDS	3′697	4′991
rRNAs (5S, 16S, 23S)	36	22
tRNAs	71	76
BUSCO score (%)	98.6	99.5

As it was reported before that *P. agglomerans* KM1 genome contained CRISPR repeats [35], the genome of *P. agglomerans* DAPP-PG 734 was checked for presence of CRISPR repeat regions with CRISPRFinder [36]. The analysis showed that the genome of *P. agglomerans* DAPP-PG 734 contained one probable CRISPR sequence on plasmid 1 and four questionable CRISPR sequences located on the chromosome and plasmid 1. As the probable CRISPR sequence only contained three spacers with a size ranging from 49 to 56 bp, and as respective *cas* genes [37] were not identified, this region most probably represents a different type of direct repeats. A re-evaluation of the CRISPR regions in the genome of *P. agglomerans* KM1 could also not confirm the presence of reliable CRISPR regions, even though this was stated by the original authors [35].

To identify and localize antibiotic resistance genes, the genome sequence of *P. agglomerans* DAPP-PG 734 was evaluated using CARD [38]. This yielded eleven potential antibiotic resistance determinants (Supplemental Table S1), of which six were transporters of either the resistance-nodulation-cell division (RND) family efflux pumps or major facilitator superfamily (MFS) efflux pumps. One putative *ampC*-type beta-lactamase with only 70% sequence identity to the *Escherichia coli ampH* beta-lactamase was found on the chromosome. Four housekeeping genes (encoding the proteins GyrB, PBP3, and both copies of EF-Tu) contained point mutations that potentially would confer antibiotic resistance to the strain.

With IslandViewer4 [39], 30 putative genomic islands (GIs) were predicted, of which the seven largest GIs were selected (Supplemental Figure S1). Genomic islands GI1—GI4 were found on the chromosome of *P. agglomerans* DAPP-PG 734 (Fig. 1). GI1 potentially represents an integrative conjugative element, while GI2 contained phage genes. GI3 and GI4 represented insertion regions with unknown function. GI5 is located on plasmid 4 and includes genes for antibiotic biosynthesis. GI6 and GI7 are found on plasmid 2. GI6 encodes for conjugative transfer proteins while GI7 contains the Hrp-1 type III secretion system (T3SS) [22].

Using PHASTER [40], one potential intact prophage region was identified in the chromosome of *P. agglomerans* DAPP-PG 734. The region (GI2, Fig. 1) has a length of 35.9 kb, is located between positions 2′701′162 and 2′737′121 (DAPPPG734_12840—DAPPPG734_13075) and has a G + C content of 53.35%. In comparison, the chromosome of *P. agglomerans* DAPP-PG 734 has a G + C content of 55.1%. Furthermore, the phage region size is similar to a 45.2 kb region of *P. vagans* C9-1 having a G + C content of 49.41%, and a 36.6 kb region of *P. agglomerans* C410P1 having a G + C content of 52.94%. The annotation of these regions all indicated that this might represent a phage.

### Comparative genomic analysis

To determine the phylogenomic relationship and to perform a pan-genome analysis for identification of genomic features, a total of 141 genomes of *P. agglomerans* and related *Pantoea* spp. were selected and integrated into a private EDGAR 3.0 database [41]. Of the genome set used in the comparisons, 122 strains were isolated from an environmental source, while only eleven isolates originated from clinical sources. For eight strains the isolation source was not indicated in the metadata of the NCBI database.

**Fig. 2:**
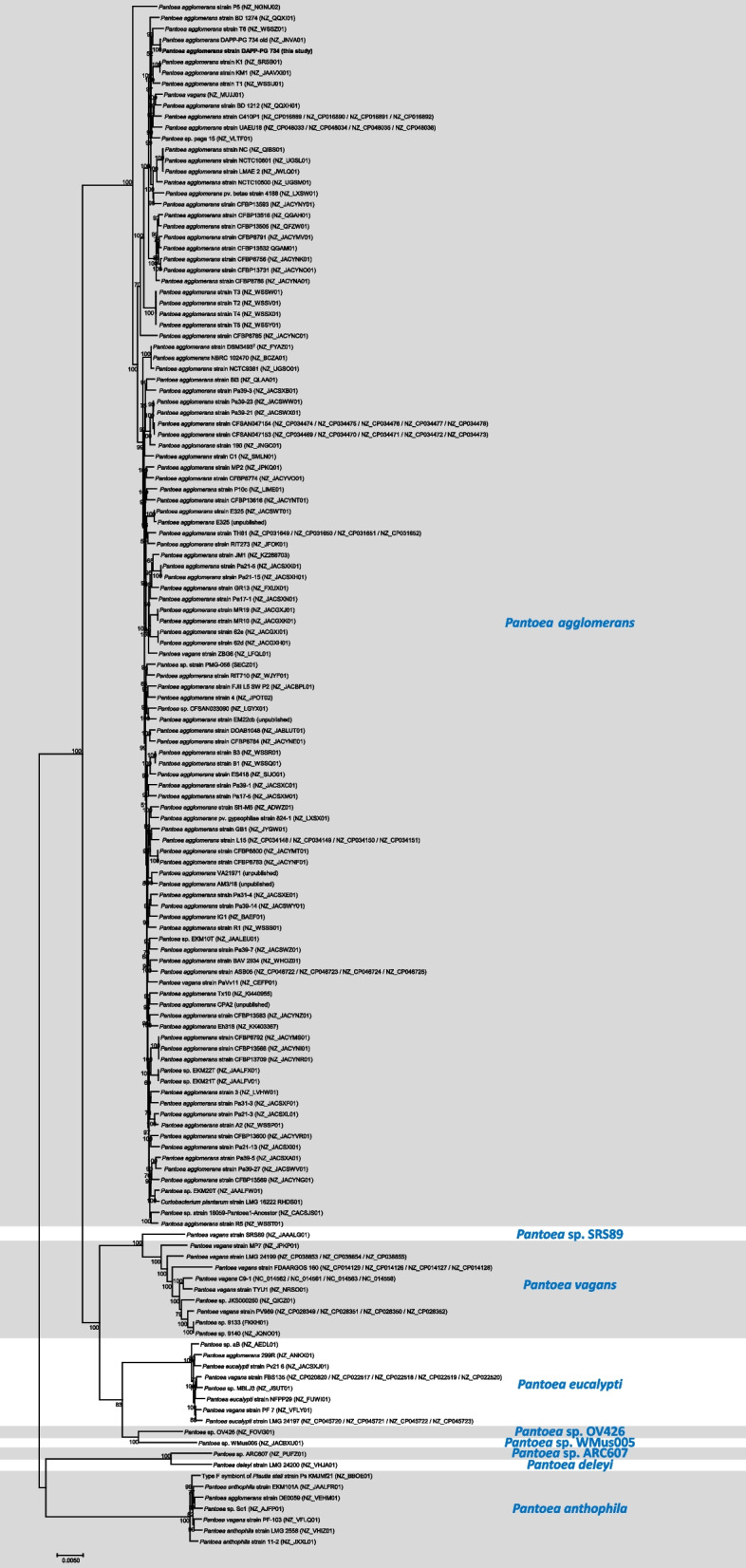
Core genome phylogenomic approximate-maximum likelihood tree of 141 *Pantoea agglomerans* and related *Pantoea* spp., constructed of a core of 1′262 genes per genome (349′521 amino acid residues per genome) computed by EDGAR 3.0*.* The accession numbers from NCBI are marked after the name of each strain. *P. agglomerans* DAPP-PG 734 is indicated in bold

The phylogenomic analysis (Fig. 2) of the core genomes of the selected strains shows that most genomes which are labelled as *Pantoea* sp. could be assigned to a known species, while other genomes were taxonomically incorrectly allocated [22]. Regarding the resulting phylogenomic tree, *P. agglomerans* DAPP-PG 734 was confirmed as a strain belonging to the species *P. agglomerans*. The allocation at species level was confirmed by using ANIb in EDGAR [41].

**Fig. 3:**
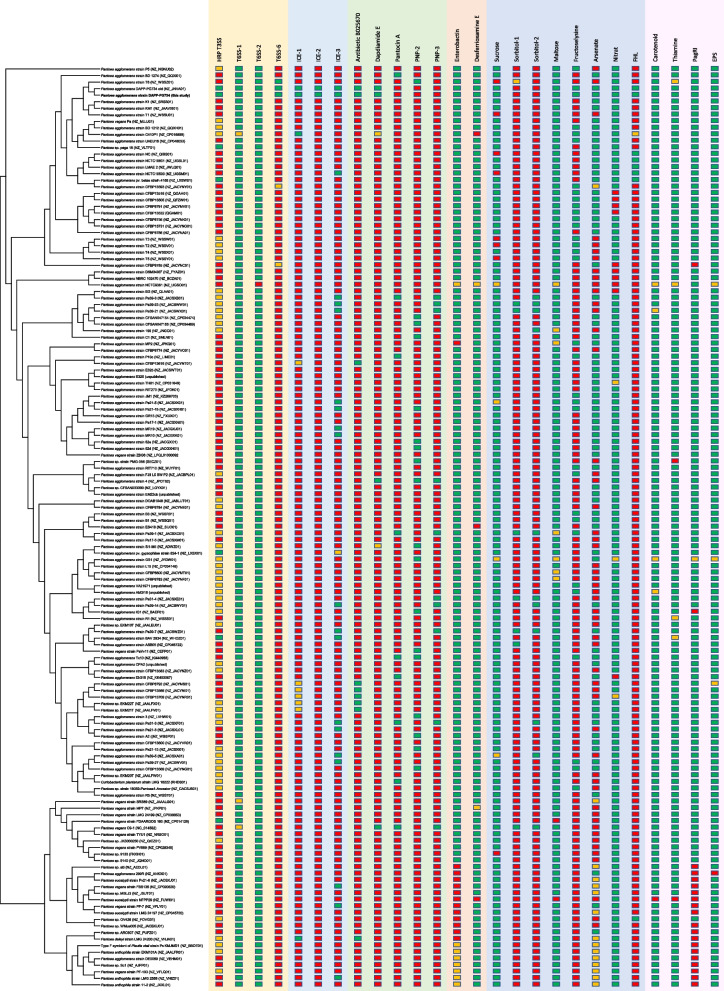
Core genome maximum likelihood phylogenetic tree of related *Pantoea* spp. genomes and their shared features. Here, all 141 strains are represented in a cladogram that only displays tree topology without branch length information (see Fig. 2) together with a table of shared features. Red squares: absence; green squares: presence; yellow squares: partial presence. Abbreviations: Hrp T3SS: type III secretion system with *hrp* genes; T6SS: type VI secretion system; ICE; integrative and conjugative element; PNP: *Pantoea* natural product; FHL: formate hydrogenlyase complex; PagRI: autoinducer quorum-sensing system regulated by N-acyl-homoserine lactone signals; EPS: exopolysaccharide biosynthesis

Using the pan-genome option in EDGAR, the genome of *P. agglomerans* DAPP-PG 734 was compared to all other strains included in the study. Differential features (Fig. 3) were checked in detail and described below.

### The LPP1 related plasmid 1 in *P. agglomerans* DAPP-PG 734

The largest plasmid in *P. agglomerans* DAPP-PG 734, plasmid 1, has a size of 530′328 bp, and represents thus close to 10% of the total genome. Related plasmids were reported before as the Large *Pantoea* Plasmid 1 (LPP-1), being present in nearly all *Pantoea* spp. [42]. Within the dataset used, the genome of only one strain, *Pantoea eucalypti* NFPP29, did not contain this plasmid (Fig. 3) and thus all features contained on it. Plasmid 1 in *P. agglomerans* DAPP-PG 734 contained a large diversity of metabolism-related genes, including the biosynthesis of carotenoids and thiamine, degradation of maltose and arabinogalactan, and uptake of iron and manganese (Supplemental Text S1). Most of these features are shared with all strains containing LPP-1 (Fig. 3) [42, 43].

Fructoselysine degradation is a relatively rare trait in *P. agglomerans* [42], and even absent in the currently available genomes of the closest related species (Fig. 3). The fructoselysine degradation gene cluster (Supplemental Figure S4) consists of four genes (*frlABDR*), while upstream of *frlA*, three additional hypothetical genes are conserved in the 33 *P. agglomerans* genomes that also contain *frlABDR*. The complete gene cluster, including the hypothetical genes, is inserted between the genes encoding for an acetyltransferase and a resolvase. Based on the observations (Supplemental Figure S4), the regions around this gene cluster are variable in their gene content.

### Plasmid 3, a second conserved plasmid in *P. agglomerans*

Plasmid 3 in *P. agglomerans* DAPP-PG 734 has a size of 163′706 bp, quite in the same range as in *P. vagans* C9-1 [44]. This plasmid belongs to a family of plasmids that is nearly ubiquitous in *P. agglomerans* and related species [45, 46]. Within our collection, there are only six genomes in which this plasmid is completely lacking, while in two strains, only parts are present. Based on our own observations with the assembly of *Pantoea* genomes [44, 46], this can, however, be due to underrepresentation of this plasmid within the read set and should be checked by mapping the original sequencing reads against a complete genome of the species [47]. Based on its ubiquity, this family of plasmids should be referred to as Large *Pantoea* Plasmid 2 (LPP-2).

For this plasmid, the only described feature is the presence of the sucrose degradation cluster (Supplemental Text S1) [45], while there are two strains that lack this feature although the plasmid is present (Fig. 3). The content of this plasmid family can thus be more variable in the different strains. However, it is not possible yet to know what the function of this plasmid is, as variants lacking this plasmid were not described yet [48].

### Plasmid 2 carrying the Hrp-1 type III secretion system

**Fig. 4:**
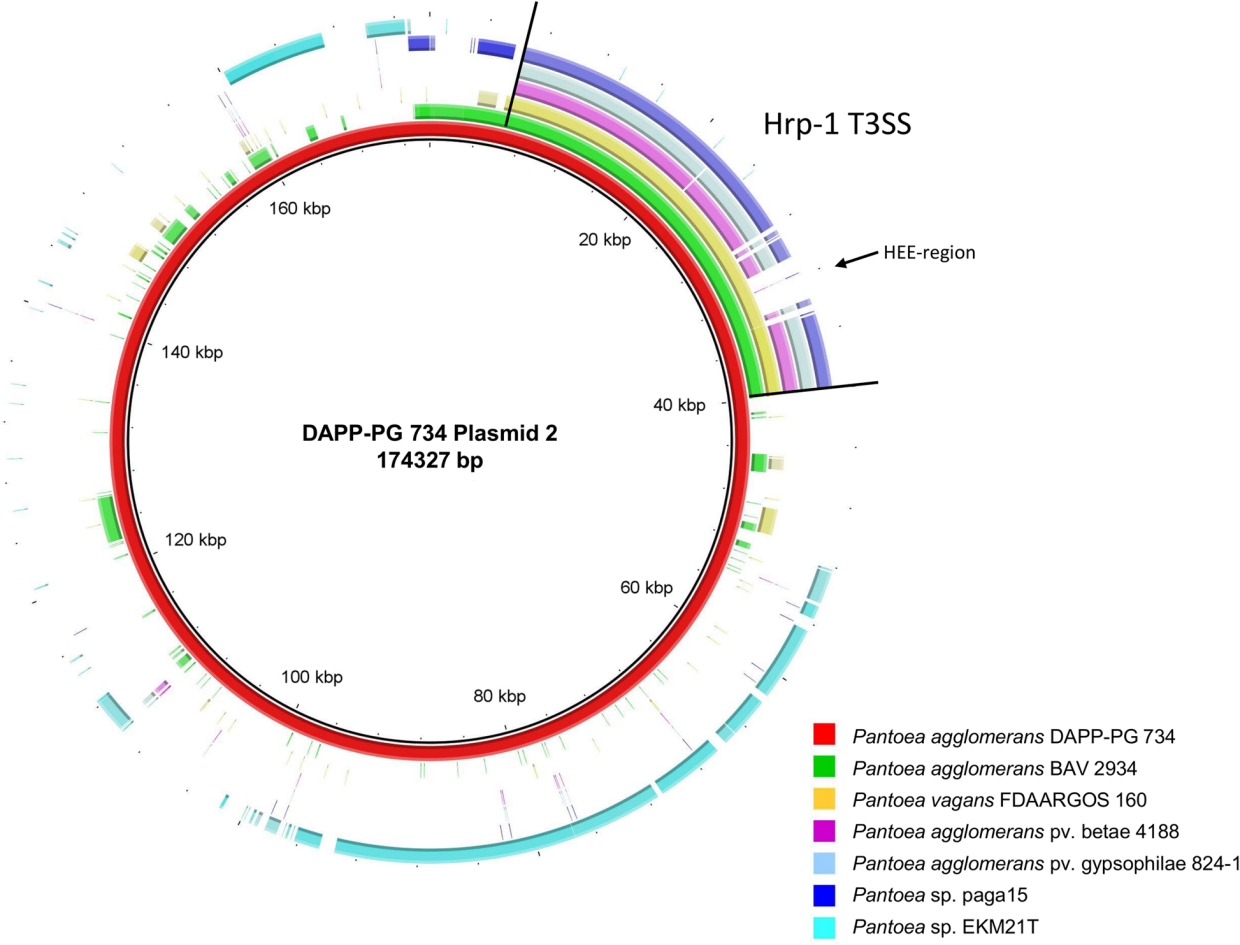
Circular genome comparison of *Pantoea agglomerans* DAPP-PG 734 plasmid 2 generated by BRIG. This figure shows a circular genome visualization of plasmid 2 of *P. agglomerans* DAPP-PG 734 in comparison with contigs containing the Hrp-1 T3SS of *P. agglomerans* BAV 2934 contig 3 (GenBank: WHOZ01000003), *Pantoea vagans* FDA-ARGOS 160 plasmid 3 (GenBank: CP014128), *Pantoea* sp. paga 15 (GenBank: VLTF01000015.1), *P. agglomerans* pv. betae 4188 contig 5 (GenBank: LXSW01000005), *P. agglomerans* pv. gypsphilae 824–1 contig 27 (GenBank: LXSX01000027) and *Pantoea* sp. EKM21T (GenBank: JAALFV010000013.1)

As already hypothesized in the previous study [22], it was confirmed from the complete genome sequence that the Hrp-1 T3SS of *P. agglomerans* DAPP-PG 734 (DAPPPG734_23535—DAPPPG734_23710) is located on a large, 174′327-bp plasmid, here called plasmid 2 (Fig. 4).

The pan-genomic analysis revealed that the gene cluster for T3SS in *P. agglomerans* DAPP PG 734 (Hrp-1 T3SS) has, next to the Hrp structural system, a complete Hrp effectors and elicitors (HEE) region [22]. This gene cluster was similar to the corresponding gene clusters of *Pantoea stewartii* subsp. *stewartii* DC283 [49] and *E. amylovora* CFBP 1430 [50, 51]. Highly similar gene cluster were present in *P. agglomerans* BAV 2934 and *P. vagans* FDA-ARGOS 160, while the gene clusters in *P. agglomerans* pv. gypsophila 824–1, *P. agglomerans* pv. betae 4188 and *P. agglomerans* paga 15, do not have a full HEE region [22].

Plasmid 2 of *P. agglomerans* DAPP-PG 734 was compared with the respective contigs containing the Hrp-1 T3SS in those strains (Fig. 4). The effector genes identified in the HEE region of the Hrp-1 T3SS of *P. agglomerans* DAPP-PG 734 are not present in the HEE region of the plant pathogens *P. agglomerans* pv. gypsophila 824–1 and *P. agglomerans* pv. betae 4188 [22]. Furthermore, it was determined that *Pantoea* sp. EKM21T, which did not contain the Hrp-1 T3SS, contained orthologs to a large fraction of genes present within plasmid 2, including several *tra* genes.

In addition, the analysis identified various types of T3SS (Hrp-2a, Hrp-2b and Hrp-3 T3SS) present in related strains, in which the genes have a different order while some genes are absent [22]. It was shown that the differential presence of T3SSs was not related to the phylogeny of the strains (Fig. 3).

### Plasmid 4, an ICE-related plasmid

**Fig. 5:**
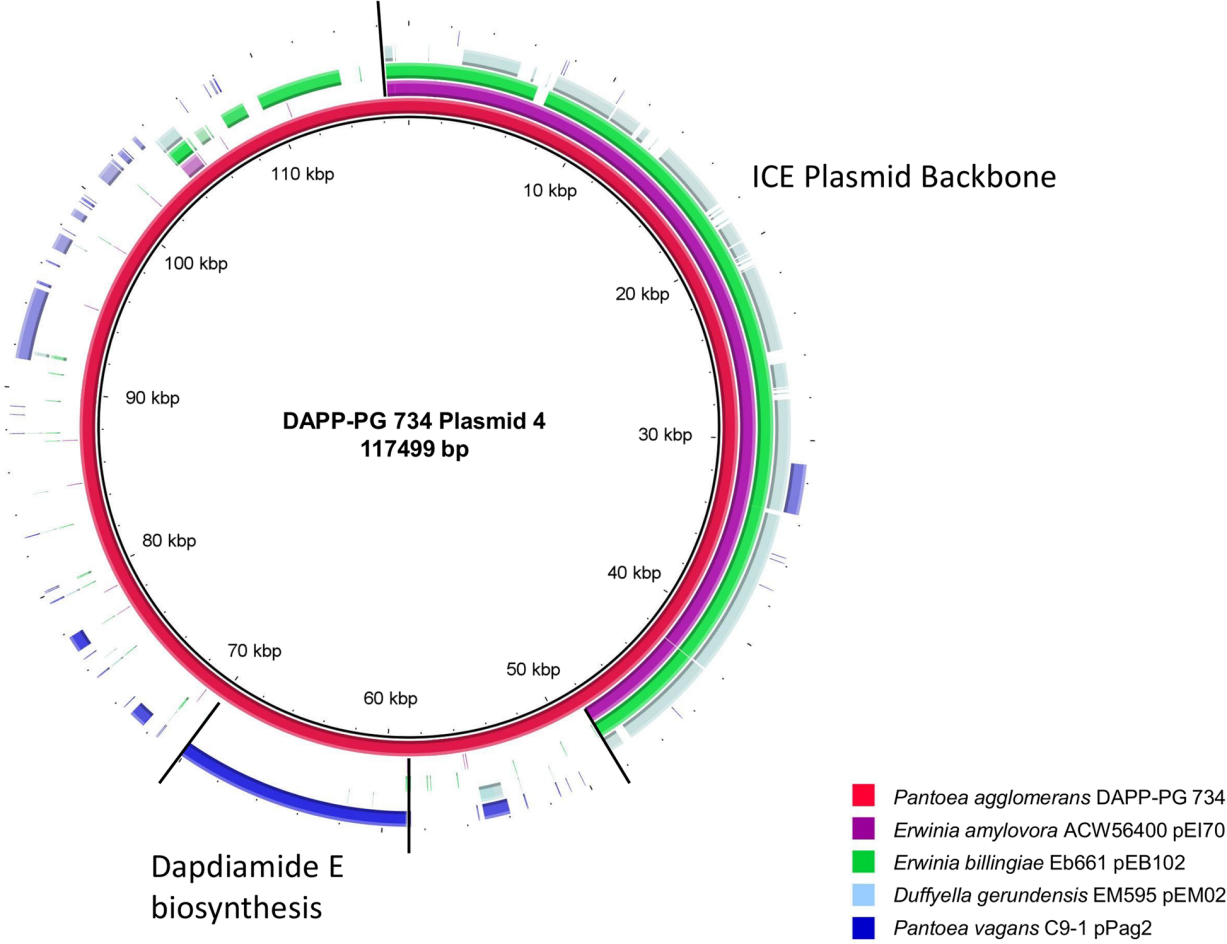
Circular genome comparison of *Pantoea agglomerans* DAPP-PG 734 plasmid 4 generated by BRIG. This figure shows a circular genome visualization of plasmid 4 of *P. agglomerans* DAPP-PG 734 in comparison with the genomic plasmids of *Erwinia amylovora* ACW56400 pEI70, *Erwinia billingiae* Eb661 pEB102, *Duffyella gerundensis* EM595^T^ pEM02 and *Pantoea vagans* C9-1 pPag2

The annotation of *P. agglomerans* DAPP-PG 734 plasmid 4, with its size of 117′499 bp the smallest plasmid in this strain, revealed that it contains a gene cluster encoding for an incomplete integrative conjugative element (Fig. 5), that shares a high similarity to ICE-based plasmid backbone regions of *E. amylovora* ACW56400 pEI70 [52], *Erwinia billingiae* Eb661 pE102 [53] and *Duffyella gerundensis* EM595^T^ pEM02 [54, 55]. This ICE region lacks several *tra* and *pil* genes and conserved genes that are common to chromosomally integrated ICEs. Additionally, pan-genome and standalone BLASTP analysis showed that the ICE-related *parA* gene and the integration and excision protein encoded by *xerC* were absent. The *attP* integration site could also not be identified. However, the plasmid contains a *repA* and plasmidborne *parAB* genes, which indicated that plasmid 4 would remain stable as a plasmid and is unable to integrate into the chromosome.

Compared to other *Pantoea* spp., the gene cluster for the plasmid-borne ICE has orthologs in 18 related strains, while it is only partly present in *P. agglomerans* pv. gypsophilae 4188 and *P. agglomerans* BAV 2934 (Fig. 3). Beside the ICE-related genes, plasmid 4 of *P. agglomerans* DAPP-PG 734 contains a large region with genes that represent cargo genes.

### Dapdiamide E biosynthesis

*P. agglomerans* DAPP PG 734 contains a nine-gene cluster for the biosynthesis of dapdiamide E [56, 57], that is located as cargo genes on plasmid 4 (DAPPPG734_25515—DAPPPG734_25555). Based on the genomic comparisons, the gene cluster of dapdiamide E was only found in four other genomes in our analysis (Fig. 3). The dapdiamide E gene cluster is present in the genome of *P. vagans* C9-1, where it is located on plasmid pPag2 [45] (Fig. 5). However, pPag2 of *P. vagans* C9-1 has nearly nothing in common with plasmid 4 of *P. agglomerans* DAPP-PG 734 (Fig. 5). The flanking regions of the gene cluster in *P. agglomerans* DAPP-PG 734 were different from all other strains (Supplemental Figure S5). This would suppose that this gene cluster was only more recently transferred to *P. agglomerans* DAPP-PG 734, although the integration method is not known.

*P. vagans* C9-1 contains two naturally separated genes which together comprise the function of *ddaF* (Supplemental Figure S5) [57]. In addition, in each of the RefSeq annotations of the clusters of *P. agglomerans* C410P1 and SL1-M5, a pseudogene was found, which could interrupt the biosynthesis of dapdiamide E, although the coding sequence is identical to the genes found in the other strains. Compared to the other 18 strains containing the ICE region of plasmid 4, the gene cluster for the biosynthesis of dapdiamide E was not found in any of these strains. In conclusion, plasmid 4 in *P. agglomerans* DAPP-PG 734 is unique as plasmid containing a gene cluster for biosynthesis of an antibiotic and gene clusters for an ICE-related plasmid.

### Biosynthesis of the antibiotic B025670

An antimicrobial biosynthesis gene cluster that is responsible for the biosynthesis of the antibiotic B025670 (DAPPPG734_18270—DAPPPG734_18335) [31] was identified in the *P. agglomerans* DAPP-PG 734. This chromosomally integrated gene cluster consists in total of 14 genes (Supplemental Figure S6), and includes eight genes encoding predicted ligases, reductase, synthases, and transferases, three genes encoding hypothetical proteins and one gene encoding a multidrug efflux pump [31]. In comparison to the gene cluster for this antibiotic in *P. agglomerans* B025670 [31], the antibiotic gene cluster in *P. agglomerans* DAPP-PG 734 was complete. It was also found in seven other strains in our data set (Fig. 3).

The gene cluster encoding for the antibiotic B025670 was indicated to be inserted between two hypothetical genes. Upstream of the antibiotic gene cluster, two genes encoding hypothetical proteins are conserved (Supplemental Figure S6). The direct flanking region was only conserved in *P. agglomerans* C410P1, while conserved regions present in all strains were quite distant. Examination of the region around the gene cluster showed that the antibiotic B025670 biosynthesis cluster is located on the genomic island GI1 (Fig. 1, Supplemental Figure S1), for which the annotation indicates that it represents an ICE. This cluster was thus probably acquired by horizontal gene transfer as well, as it was identified as so-called cargo genes in the ICE region.

### Type VI secretion systems

Type VI secretion systems (T6SS) are described as injectosome-like molecule constructions localized between the cytoplasmic membrane and the outer membrane of the T6SS-producing bacteria [58]. However, T6SSs are used to have a significant advantage of bacterial fitness in competition with other environmental microorganisms by transporting toxic proteins across the membrane of the target organism. This secretion system is constituted by 15–25 different proteins, including 13 core genes encoding conserved components which are necessary for T6SS [59, 60].

As in several other *Pantoea* spp. [59], *P. agglomerans* DAPP-PG 734 contains the conserved T6SS-1 and T6SS-2. The gene cluster of T6SS-2 was identical to other related strains and was identified in all other strains (Fig. 3). The gene cluster of T6SS-1 showed a similar structure as described for *P. agglomerans* E325 [59]. However, differences between the strains can be recognized in the variable regions around the *hcp* gene and downstream of the gene cluster after the first *vgrG* gene (Supplemental Figure S7). It thus shows that next to interspecies differences [59], also intraspecies differences occur.

In the genome of *P. agglomerans* DAPP-PG 734, an additional T6SS was identified (DAPPPG734_15850 – DAPPG734_15980), which we named here, in analogy to the earlier study, T6SS-6, as this cluster type was not observed in *Pantoea* spp. before. The additional T6SS-6 in *P. agglomerans* DAPP-PG 734 was only found in three other strains analyzed in this study (Fig. 3). The gene cluster for T6SS-6 contains the conserved genes *hcp* and *vgrG* as well as part of the *tss* genes, including *tssBCFGHJKLM*. In the Refseq annotation at NCBI, the two strains *P. agglomerans* CFBP 8785 and CFBP 13,593 have a pseudogene in one of the genes downstream of the gene T6SS-6 cluster, but with identical sequence as in the other strains (Supplemental Figure S8). This may indicate that RefSeq in some cases would annotate genes spuriously as pseudogenes. The whole region of T6SS-6 is inserted between the gene *pucI* encoding for putative allantoin permease and a tRNA-Asp. However, no evidence was found on how this cluster was inserted at this position.

### Iron acquisition

Iron is important as cofactor in proteins in almost every living microorganism. To take up and utilize iron from the environment, bacteria are producing high-affinity uptake systems. Microorganisms can synthesize small molecules (400–1000 Da), called siderophores, which complex iron ions and increase the bioavailability of total iron [61]. The biosynthesis of a siderophore can play an important role as a biocontrol trait by competition with phytopathogens for the already limited supply of iron in plants [62, 63]. The genome of *P. agglomerans* DAPP-PG 734 contains the biosynthetic genes for the hydroxamate siderophore desferrioxamine E (*dfoJACS*) and the catecholate siderophore enterobactin (*ent-fep*).

The *dfoJACS* gene cluster (DAPPPG734_21835—DAPPPG734_21850) [63–65] is located on plasmid 1 and consists of four genes which are responsible for the biosynthesis. It was shown that *P. agglomerans* DAPP-PG 734 produced diffusible siderophores on chrome azurol S (CAS) agar plates [22], indicating that desferrioxamine E is produced. In comparison to related strains, the *dfoJACS* gene cluster is present in almost all related *Pantoea* spp., except for *P. eucalypti* NFPP29, which does not contain the LPP-1 related to plasmid 1 of *P. agglomerans* DAPP-PG 734.

In contrast, the *ent-fep* gene cluster [65, 66] contains 12 genes (Supplemental Figure S9) and is located on the chromosome (DAPPPG734_17780—DAPPPG734_17845). The *ent-fep* gene cluster is absent in all strains of the species *P. eucalypti*, *Pantoea* sp. OV426, *Pantoea* sp. ARC607 and *P. deleyi* (Fig. 3). Furthermore, all members of the species *Pantoea anthophila* lacked the *entS* gene. This gene encodes for an MFS pump for the secretion of enterobactin [67]. The loss of thereof does not have a significant impact on the growth under iron-limited conditions as enterobactin can still be modified and transported out of the cell in its glycosylated form [68].

### Autoinducer biosynthesis

QS systems plays a main role in virulence of plant pathogenic bacteria in response to cell density by regulating gene expression along production and detection of signal molecules, commonly an AHL [69]. AHLs in *Pantoea agglomerans* are produced by an AHL synthase PagI, which is then formed to a complex by a transcriptional regulator PagR [19, 70]. The mechanism of autoinducer QS system regulated by AHL signals has been well described in the plant pathogen *P. stewartii* subsp. *stewartii* DC283 [69] and has been reported in the gall-forming bacterium *P. agglomerans* pv. gypsophilae 824.1 [19] and in the pathogen *P. ananatis* [71]. The *pagRI* gene cluster was identified in *P. agglomerans* DAPP-PG 734 on the chromosome (DAPPPG734_17300—DAPPPG734_17305), in contrast to *P. vagans* C9-1, where it is located on pPag3 [10, 43].

The genome comparisons showed that the genes *pagRI* were absent in *P. agglomerans* IG1 and *P. agglomerans* CFBP 8785 (Supplemental Figure S10). Additionally, it was not detected in the species *P. eucalypti*, *Pantoea* sp. WMus005, *Pantoea* sp. ARC607, *P. deleyi* and *P. anthophila* (Fig. 3), indicating that the *pagRI* genes are only present in *P. agglomerans*, *Pantoea* sp. OV426 and *P. vagans* [10, 72]. Compared to related strains, the gene region between *cdh* and *symE* varies by the presence of additional genes (Supplemental Figure S10). The region of *P. agglomerans* CFBP 8785 was not included in the figure as, due to a larger deletion in the region, the *symE* gene is not present anymore.

### Exopolysaccharide biosynthesis

*P. agglomerans* strains produce an exopolysaccharide (EPS) similar to the high-molecular weight heteropolysaccharide stewartan produced by *P. stewartii* subsp. *stewartii* [73]. A 12-gene *cps* region (Supplemental Figure S11) is responsible for this biosynthesis [74]. Additionally, an activation signal sensing phosphorelay system encoded by *rcsABC* genes is required [75]. *P. agglomerans* DAPP-PG 734 contains the complete gene cluster for the biosynthesis of the stewartan-like EPS (DAPPPG734_06845—DAPPPG734_06910) (Supplemental Figure S11). Additional genes encoding for diverse mannosyltransferases and glycosyltransferases were identified downstream of the EPS cluster, which could have an impact on the biosynthesis of EPS. Based on the comparison to other strains, the regions containing additional genes were very variable (Supplemental Figure S11). The activator genes *rcsA* (DAPPPG734_07900), *rcsB* (DAPPPG734_06370) and *rcsC* (DAPPPG734_07685) are spread over different locations on the chromosome. Based on the pan-genome analysis, detailed studies of the gene content for EPS biosynthesis, coupled to structural analysis of the EPS produced by each strain [76], would be necessary, as more potentially EPS biosynthesis gene clusters were identified.

### Anaerobic formate metabolism

Formate is the signature compound in the anaerobic mixed acid-fermentative metabolism of *E. coli* and other enterobacteria. The gene cluster for biosynthesis and mature process of formate hydrogenlyase (FHL) complex [77] was identified on the chromosome of *P. agglomerans* DAPP-PG 734 (DAPPPG734_10275—DAPPPG734_10365) and in that of seven other strains (Fig. 3), indicating that it is rather a rare trait within the species. The FHL gene cluster is inserted between the *pfkA* gene and the *yceI* gene. Variation within the cluster was not observed, indicating the conserved structure of this gene cluster. *P. agglomerans* C410P1 is the only strain containing a potential pseudogene within the gene cluster of the FHL complex, although the sequence is identical to that of the orthologs.

### The ecological niche of *P. agglomerans* DAPP-PG 734

The aim of this study was to examine key genomic features in the genome of *P. agglomerans* DAPP-PG 734. Based on the genome sequence of the strain, which was isolated from olive knots [32], we can now use the identified features to hypothesize about its potential ecological role. In this study, we found several gene clusters encoding different secretion systems, especially Hrp-1 T3SS, which plays an important role in suppressing the host defense system for synergistic effects with *P. savastanoi* pv. savastanoi. A recent study [22] showed that *P. savastanoi* pv. savastanoi DAPP-PG 722 and *P. agglomerans* DAPP-PG 734 form a stable interspecies community to proliferate in olive knots. However, the study also demonstrated that *P. savastanoi* pv. savastanoi DAPP-PG 722 colonies were localized around those of *P. agglomerans* DAPP-PG 734 within the knots [22], while *Erwinia toletana* DAPP-PG 735 required the proximity of *P. savastanoi* pv. savastanoi for its survival and growth in olive knots [20, 78]. This vicinity did not occur with *P. agglomerans* DAPP-PG 734, which was hypothesized to be based on the biosynthesis of differential AHLs [32]. This study rather showed that the position within the olive knots may be based on the sensitivity of *P. savastanoi* pv. savastanoi DAPP-PG 722 to the antibiotics produced by *P. agglomerans* DAPP-PG 734.

In combination with a *P. savastanoi* strain, *Pantoea agglomerans* paga was able to increase the percentage of knots in one olive cultivar [79]. This strain alone did not cause knots on olive trees. The genome of this strain does, in contrast to *P. agglomerans* DAPP-PG 734, not contain antibiotic biosynthesis genes (Fig. 3), but it does contain a more similar *Hrp*-1 gene cluster and some type 3 secretion system effectors as in the plant pathogens *P. agglomerans* pv. betae strain 4188 and *P. agglomerans* pv. gypsophilae strain 824–1 [22]. The effect of the genetic setup of the two strains should thus be tested in parallel to understand the role of these systems during olive knot formation.

In conclusion, *P. agglomerans* DAPP-PG 734, as a single endophyte, does not harm olive trees and does not induce any consequent knot formation [22], in contrary, it is even assumed that the strain helps the tree to repress and suppress pathogenic bacteria by producing antibacterial substances. Subsequently, *P. savastanoi* pv. savastanoi may take advantage of the available space to grow around *P. agglomerans* DAPP-PG 734 and can start knot formation in olive trees. Furthermore, the presence of two gene clusters for the biosynthesis of dapdiamide E and antibiotic B025670 in *P. agglomerans* DAPP-PG 734 could indicate that this strain has the potential as a biocontrol agent against other plant pathogens as well.

## Conclusion

In this study, we examined the factors giving indications to the potential role of *P. agglomerans* DAPP-PG 734 as endophyte in olive knots. For this reason, the genome of *P. agglomerans* DAPP-PG 734 was completed to be able to study genomic features in more detail, which was only limited possible with the draft genome [22]. Strain-specific gene clusters were discovered and identified using comparative genomics. However, a good genomic comparison requires that the sequenced genomes of closely related strains are of a high quality as well [47].

Based on the genomic analysis, we can assume that *P. agglomerans* DAPP-PG 734 is playing an important role as endophyte and not as pathogen in the formation of olive knot disease. As a single endophyte, *P. agglomerans* DAPP-PG 734 does not harm olive trees at all, while in presence of plant pathogens, it represses the growth thereof by producing antimicrobial substances. Due to the available space, *P. savastanoi* pv. savastanoi takes advantage to proliferate and induce olive knot formation enhanced by the active Hrp-1 T3SS of *P. agglomerans* DAPP-PG 734. Furthermore, the presence of ecologically relevant genomic clusters for biosynthesis of two different antibiotic metabolites, several secretion systems (including the T6SSs) and an autoinducer indicate that *P. agglomerans* DAPP-PG 734 may rather be involved as biocontrol organism against plant pathogens. Nevertheless, future research should be performed to confirm the biosynthesis of these antibiotics in vitro and in vivo. Furthermore, additional research will be necessary to understand the role of *P. agglomerans* DAPP-PG 734 as an endophytic bacterium in a plant pathogenic community and the interaction with other plant pathogens resulting in disease formation.

## Methods

### Genome sequencing, assembly and annotation

To improve the genome assembly of *P. agglomerans* DAPP-PG 734 [21], the genome was resequenced with MinION long read sequencing. Genomic DNA was extracted from *P. agglomerans* DAPP-PG 734 grown overnight at 28 °C with 220 rpm shaking in LB medium using the Gentra PureGene Yeast/Bact kit according to the manufacturer’s protocol (Qiagen, Hilden, Germany). The genomic DNA was quantified using the Quant-iT PicoGreen double-stranded DNA quantification assay (Thermo Fisher Scientific, Waltham, MA) and the quality was checked by using a fragment analyzer (Advanced Analytical Technologies, Inc., Ankeny, IA). For library preparation and sequencing, the ligation sequencing kit (catalog no. SQKLSK109; Oxford Nanopore Technologies, Oxford, United Kingdom) and a MinION sequencer equipped with a R9.4.1 Flongle flow cell were used. Multiplexing was performed by using the native barcoding expansion kit (catalog no. XP-NBD114) and base calling was performed using Guppy v.3.3.3 (Oxford Nanopore Technologies).

After sequencing, a first hybrid assembly with Unicycler v.0.4.8 [33] was performed using the Illumina MiSeq reads determined before [22] and the MinION reads. After the first assembly, a manual assembly was performed to check misalignments and to improve the assembly of the genome sequence of *P. agglomerans* DAPP-PG 734 [47]. SeqMan NGen 12.0 (DNASTAR, Madison, WI, USA) was used to map the Illumina reads against the draft genome. Additionally, SeqMan Pro 12.0 (DNASTAR) was used for manual checking to uncover assembly errors. Throughout the assembly procedure, EditSeq 12.0 (DNASTAR) was used as supplemental software to edit the genome sequence. After the complete genome sequence was available as a final FastA file, annotation was performed with the software program Bakta v.1.2.4 [80]. The genome was submitted to the EMBL/ENA repository and received accession numbers OW970315-OW970319 for chromosome and plasmids 1 to 4, respectively.

### Comparative genomics

Comparative genomics was performed with the annotated genome sequence of *P. agglomerans* DAPP-PG 734 and a group of 140 *P. agglomerans* and related genomes. For the comparisons, a comparative pan-genome analysis with the program EDGAR 3.0 [41] was performed. To infer the phylogenetic relations between the strains, a phylogenetic tree was constructed by computing the core genomes of the chosen strains in EDGAR 3.0 [41]. For this, multiple alignments of each orthologous gene set were generated using the program MUSCLE [81]. The output was concatenated and used to generate an approximately-maximum-likelihood phylogenetic tree using the open-source program FastTree [82]. By using the Shimodaira-Hasegawa test, FastTree computes local support values to quickly estimate the reliability of each division in the tree. To present the phylogenomic tree as an image, the resulting tree was edited within the software MEGA X [83]. For species differentiation, average nucleotide identity (ANI) was applied as integrated program in EDGAR [41]. Specific genomic islands and clusters of interest from the pan-genome analysis were manually selected and analyzed with BLAST when required. Furthermore, for visualization, cluster figures were generated with several subroutines of the Lasergene Package v5 (DNASTAR).

In addition, the progressive MAUVE algorithm in MAUVE 3.1.2 [84] was used to sort all contigs and to visualize the genome alignments of *P. agglomerans* DAPP-PG 734 with the complete genomes of *P. agglomerans* C410P1 (GenBank: CP016889 / CP016890 / CP016891 / CP016892), *P. agglomerans* CFSAN047154 (GenBank: CP034474 / CP034475 / CP034476 / CP034477 / CP034478), *P. agglomerans* L15 (GenBank: CP034148 / CP034149 / CP034150 / CP034151) and *P. vagans* C9-1 (GenBank: CP002206 / CP001893 / CP001894 / CP001895). Circular plasmid images were created using BLAST Ring Image Generator (BRIG v. 0.95) [85]. Clustered regularly interspaced short palindromic repeats (CRISPR) were identified by using the web version of CRISPRFinder [36]. The online software Phage Search Tool Enhanced Release (PHASTER) [40, 86] was used to find potential prophages. Putative genomic islands were predicted by using the prediction methods IslandPath-DIMOB, SIGI-HMM, and IslandPick in the web version IslandViewer4 [39] while the genome sequence of DAPP-PG 734 was aligned against the reference genome of *P. vagans* C9-1 [44]. To predict antibiotic resistance genes (ARG), the online tool Resistance Gene Identifier (RGI) v.5.2.0 in the Comprehensive Antibiotic Resistance Database (CARD) v.3.1.4 [38, 87] was used. In a further step, the resulting reference sequences from RGI were compared against the genome sequence of *P. agglomerans* DAPP-PG 734 with BLAST at NCBI to identify the exact location.

## Supplementary Information

**Additional file 1:** **Text S1.** Metabolic versatility in *Pantoea agglomerans* DAPP-PG 734. **Table S1.** Antibiotic resistance gene profile in the genome of *Pantoea agglomerans* DAPP-PG 734. **Figure S1.** Genomic islands in *Pantoea agglomerans* DAPP-PG 734.** Figure S2.** MAUVE alignment of *Pantoea agglomerans* DAPP-PG 734 plasmid 1 and Pantoea vagans C9-1 pPag3. **Figure S3.** MAUVE alignmentof *Pantoea agglomerans* DAPP-PG 734 plasmid 3 and *Pantoea vagans* C9-1 pPag1.** Figure S4.** Gene cluster for degradation of fructoselysine in five strains of *Pantoea agglomerans*. **Figure S5.** Gene cluster for biosynthesis of dapdiamide E in five *Pantoea* spp. **Figure S6.** Gene cluster for biosynthesis of antibiotic B025670 in six *Pantoea* spp. **Figure S7.** Gene cluster for type VI secretion system 1 (T6SS-1) in five *Pantoea* spp. **Figure S8.** Gene cluster for type 6 secretion system 6 (T6SS-6) in five *Pantoea agglomerans*. **Figure S9.** Gene cluster for biosynthesis of enterobactin in four *Pantoea* spp. **Figure S10.** Gene cluster of the autoinducer biosynthesis *pagRI* in six *Pantoea* spp. **Figure S11.** Gene cluster for biosynthesis of exopolysaccharide (EPS) in four *Pantoea agglomerans*.

## Data Availability

Genomic sequences generated in this study have been submitted to EMBL and received the accession numbers OW970315-OW970319 for chromosome and plasmids 1 to 4, respectively. The authors also declare that there is no conflict of interest to disclose.
