# Supplementary material for: Comparative genomics to examine the endophytic potential of Pantoea agglomerans DAPP-PG 734
Source: BMC Genomics. 2022 Nov 8;23:742. doi: 10.1186/s12864-022-08966-y (PMC9641835; doi:10.1186/s12864-022-08966-y)
Supplement: Supplementary file 1 — Additional file 1: Text S1. Metabolic versatility in Pantoea agglomerans DAPP-PG 734. Table S1. Antibiotic resistance gene profile in the genome of Pantoea agglomerans DAPP-PG 734. Figure S1. Genomic islands in Pantoea agglomerans DAPP-PG 734. Figure S2. MAUVE alignment of Pantoea agglomerans DAPP-PG 734 plasmid 1 and Pantoea vagans C9-1 pPag3. Figure S3. MAUVE alignmentof Pantoea agglomerans DAPP-PG 734 plasmid 3 and Pantoea vagans C9-1 pPag1. Figure S4. Gene cluster for degradation of fructoselysine in five strains of Pantoea agglomerans. Figure S5. Gene cluster for biosynthesis of dapdiamide E in five Pantoea spp. Figure S6. Gene cluster for biosynthesis of antibiotic B025670 in six Pantoea spp. Figure S7. Gene cluster for type VI secretion system 1 (T6SS-1) in five Pantoea spp. Figure S8. Gene cluster for type 6 secretion system 6 (T6SS-6) in five Pantoea agglomerans. Figure S9. Gene cluster for biosynthesis of enterobactin in four Pantoea spp. Figure S10. Gene cluster of the autoinducer biosynthesis pagRI in six Pantoea spp. Figure S11. Gene cluster for biosynthesis of exopolysaccharide (EPS) in four Pantoea agglomerans. [file 12864_2022_8966_MOESM1_ESM.pdf]

Supplemental Material

belonging to:

Comparative genomics to examine the endophytic potential of *Pantoea agglomerans* DAPP-PG 734

Arburon Sulja, Joël F. Pothier, Jochen Blom, Chiaraluce Moretti, Roberto Buonaurio, Fabio Rezzonico and Theo H. M. Smits

Content:

Text S1: Metabolic versatility in *Pantoea agglomerans* DAPP-PG 734.

Table S1: Antibiotic resistance gene profile in the genome of *Pantoea agglomerans* DAPP-PG 734.

Figure S1: Genomic islands in *Pantoea agglomerans* DAPP-PG 734.

Figure S2: MAUVE alignment of *Pantoea agglomerans* DAPP-PG 734 plasmid 1 and *Pantoea vagans* C9-1 pPag3.

Figure S3: MAUVE alignment of *Pantoea agglomerans* DAPP-PG 734 plasmid 3 and *Pantoea vagans* C9-1 pPag1.

Figure S4: Gene cluster for degradation of fructoselysine in five strains of *Pantoea agglomerans*.

Figure S5: Gene cluster for biosynthesis of dapdiamide E in five *Pantoea* spp.

Figure S6: Gene cluster for biosynthesis of antibiotic B025670 in six *Pantoea* spp.

Figure S7: Gene cluster for type VI secretion system 1 (T6SS-1) in five *Pantoea* spp.

Figure S8: Gene cluster for type 6 secretion system 6 (T6SS-6) in five *Pantoea agglomerans*.

Figure S9: Gene cluster for biosynthesis of enterobactin in four *Pantoea* spp.

Figure S10: Gene cluster of the autoinducer biosynthesis *pagRI* in six *Pantoea* spp.

Figure S11: Gene cluster for biosynthesis of exopolysaccharide (EPS) in four *Pantoea agglomerans*.

Supplemental references

Text S1: Metabolic versatility in *Pantoea agglomerans* DAPP-PG 734.

The plasmids 1 and 3 in *P. agglomerans* DAPP-PG 734 carries several gene clusters as most were observed in *P. vagans* C9-1 on pPag1 and pPag3 [1, 2] for metabolic purpose and biosynthesis of secondary metabolites (**Figure S2, Figure S3**). However, the plasmids from DAPP-PG 734 are also containing some variation compared to the plasmids of C9-1.

Plasmid 1 include a complete gene cluster (*malGFEKLMQPT*) for maltose metabolism (DAPPPG734_22555 - DAPPPG734_22605). The maltose/maltodextrin system is regulated by an activator MalT, and contains eight further regulated genes, which are shaped for the transport and metabolism of maltose and maltodextrins. Six genes of this cluster encode a high-affinity and binding protein-dependent ABC transporter, called maltoporin, MalM, MalE, MalK and the subunits MalF and MalG [3]. Compared to related strains (**Figure 3, main document**), only two strains showed the absence of the *malGFEKLMQPT* gene cluster. *Pantoea eucalypti* NFPP29 does not contain any plasmids related to plasmid 1 in *P. agglomerans* DAPP-PG 734 or pPag3 in *P. vagans* C9-1 [2]. On the other hand, *P. vagans* FDAARGOS_160 contains a plasmid related to plasmid 1 in DAPP-PG 734, but it lacks all *mal* genes.

Other clusters for carbohydrate metabolism including arabinogalactan (*ganKEFGABCLR*) [4](DAPPPG734_23085 - DAPPPG734_23130) and fructoselysine (*frlABDR*) (DAPPPG734_21510 - DAPPPG734_21525) are located on plasmid 1 as observed in a large group of *Pantoea* strains which include a large universal *Pantoea* plasmid (LPP-1)[5]. The *frlABDR* gene cluster consist of four genes encoding for fructoselysine permease FrlA, fructoselysine-6-phosphate deglycase FrlB, fructoselysine-6-phosphatase FrlD and regulator of fructoselysine operon FrlR (**Figure S4**). The presence of the *frlABDR* gene cluster is very variable within the compared strains (**Figure 3, main document**). This indicated that *frlABDR* is rather a variable trait within the species [5]. Therefore, further analyses are necessary to determine the role of this gene cluster within the species.

Corresponding to the production of the typically yellow pigment [6], *P. agglomerans* DAPP-PG 734 contains six genes (*crtEXYIBZ*) for carotenoid biosynthesis as observed in *P. vagans* C9-1 on pPag3 [2], which are located on plasmid 1 (DAPPPG734_22760 - DAPPPG734_22785). Carotenoids can play an important role in photooxidative damage protection and protection against environmental stress [7]. The *crtEXYIBZ* gene cluster were identified in almost all other strains (**Figure 3, main document**), except *P. eucalypti* NFPP29, which does not have a plasmid related to plasmid 1 in *P. agglomerans* DAPP-PG 734.

Furthermore, the gene cluster for thiamine biosynthesis is also present in DAPP-PG 734 on plasmid 1 (DAPPPG734_22825 - DAPPPG734_22840) and consists of four genes (*thiOSGF*) as discovered in *P. vagans* C9-1 on pPag3 [2]. Recent results showed that the biosynthesis of thiamine has an impact of enhancing the biosynthesis of exopolysaccharides (EPS) in *Erwinia amylovora* which cause

necrotrophic fire blight disease of apple, pear and other rosaceous plants [8]. In comparison, the gene cluster *thiOSGF* was only absent in *Pantoea* sp. PMG_056 and *P. eucalypti* NFPP29 (**Figure 3, main document**).

A gene cluster for encoding a heavy metal reduction of arsenate is also found on the plasmid 1 in DAPP-PG 734 (DAPPPG734_22475 - DAPPPG734_22490). In addition, some *P. agglomerans* strains contain an *arsH* gene encoding a putative flavoprotein [9]. The presence of the *arsCBRH* gene cluster is also here very variable within the compared strains (**Figure 3, main document**), indicating that it is rather a variable trait within the species [9]. It is important to mention that most strains do not include the gene *arsH* and were therefore marked as partially containing the gene cluster. Additional research *in vitro* will be necessary to indicate the diversification and presence of heavy metal reduction within the species. A further gene cluster for inner membrane iron and manganese transporter (*sitABCD*) as observed on LPP-1 [5] is also located on plasmid 1.

Based on the genomic analysis, plasmid 3 includes only a complete gene cluster for sucrose metabolism (DAPPPG734_25120 - DAPPPG734_25140) [1, 10]. Sucrose is the most common disaccharide energy source for phytopathogenic bacteria and consist of a glucose unit linked to a fructose unit through a glycosidic linkage [11]. Four structural genes (*scrABKY*) are responsible for the transport and utilization of sucrose, which includes an ATP-dependent fructokinase, a sucrose-specific porin of the outer membrane, an enzyme of the phosphoenolpyruvate-dependent phosphotransferase system (PTS), and beta-fructofuranosidase fructohydrolase for cleaving sucrose 6-phosphate into alpha-glucose 6-phosphate and beta-fructose. The sucrose metabolic system is regulated by a sucrose operon repressor (ScrR) and is induced in a sucrose-specific manner [12]. In comparison, the *scrABKY* gene cluster was only absent in eight other strains (**Figure 3, main document**). These strains do not contain a plasmid related to plasmid 3 in *P. agglomerans* DAPP-PG 734 or pPag1 in *P. vagans* C9-1 [2].

Genes for sorbitol metabolism as observed in *P. vagans* C9-1 on pPag2 [1] were absent in DAPP-PG 734. Additionally, *P. agglomerans* DAPP-PG 734 contains a gene cluster (*narIJHGK*) for the reduction of nitrate to nitrite which can act as terminal respiratory electron acceptor [13]. This gene cluster is located on the chromosome (DAPPPG734_11365 - DAPPPG734_11390) directly adjacent to the T6SS-2. A gene for encoding flavorubredoxin is also located in this nitrate reductase metabolism cluster. Flavorubredoxin acts like a reductase partner of the anaerobic nitric oxide reductase where nitric oxide is detoxified by using NADH and flavorubredoxin [14]. Compared to related strains, only ten other strains lack all *nar* genes (**Figure 3, main document**).

Table S1: Antibiotic resistance gene profile in the genome of *Pantoea agglomerans* DAPP-PG 734 as predicted using CARD [15].

Antibiotic Resistance Genes (ARG)	SNP	AMR gene family	Drug class	Resistance mechanism	% Identity	Location	Locus tag
crp		resistance-nodulation-cell division (RND) antibiotic efflux pump	macrolide antibiotic, fluoroquinolone antibiotic, penam	antibiotic efflux	98.57	Chromosome	DAPPPG734_02050
rsmA		resistance-nodulation-cell division (RND) antibiotic efflux pump	fluoroquinolone antibiotic, diaminopyrimidine antibiotic, phenicol antibiotic	antibiotic efflux	89.66	Chromosome	DAPPPG734_16755
Klebsiella pneumoniae kpnH		major facilitator superfamily (MFS) antibiotic efflux pump	macrolide antibiotic, fluoroquinolone antibiotic, aminoglycoside antibiotic, carbapenem, cephalosporin, penam, peptide antibiotic, penem	antibiotic efflux	88.02	Chromosome	DAPPPG734_04800
Escherichia coli ampH beta-lactamase		ampC-type beta-lactamase	cephalosporin, penam	antibiotic inactivation	70.54	Chromosome	DAPPPG734_05630
Klebsiella pneumoniae kpnF		major facilitator superfamily (MFS) antibiotic efflux pump	macrolide antibiotic, aminoglycoside antibiotic, cephalosporin, tetracycline antibiotic, peptide antibiotic, rifamycin antibiotic	antibiotic efflux	75.73	Chromosome	DAPPPG734_08635
adeF		resistance-nodulation-cell division (RND) antibiotic efflux pump	fluoroquinolone antibiotic, tetracycline antibiotic	antibiotic efflux	60.8	Chromosome	DAPPPG734_10495
adeF		resistance-nodulation-cell division (RND) antibiotic efflux pump	fluoroquinolone antibiotic, tetracycline antibiotic	antibiotic target alteration	42.06	Chromosome	DAPPPG734_15020
Morganella morganii gyrB conferring resistance to fluoroquinolones	S4363	fluoroquinolone resistant gyrB	fluoroquinolone antibiotic	antibiotic target alteration	80.85	Chromosome	DAPPPG734_00025
Haemophilus influenzae PBP3 conferring resistance to beta-lactam antibiotics	D350N, S357N	Penicillin-binding protein mutations conferring resistance to beta-lactam antibiotics	cephalosporin, cephamycin, penam	antibiotic target alteration	53.19	Chromosome	DAPPPG734_16625
Escherichia coli EF-Tu mutants conferring resistance to pulvomycin	R234F	elfamycin resistant EF-Tu	elfamycin antibiotic	antibiotic target alteration	91.09	Chromosome	DAPPPG734_02175
Escherichia coli EF-Tu mutants conferring resistance to pulvomycin	R234F	elfamycin resistant EF-Tu	elfamycin antibiotic	antibiotic target alteration	89.57	Chromosome	DAPPPG734_20110

Figure S1: Genomic islands in *Pantoea agglomerans* DAPP-PG 734. This figure shows the circular plot of the genome of *P. agglomerans* DAPP-PG 734 and the predicated genomic islands which are colored based on the prediction methods [16]. Orange indicates genomic islands predicted by SIGI-HMM, blue represent genomic islands using IslandPath-DIMOB method and red shows the genomic islands that were predicted by an integrated analysis.

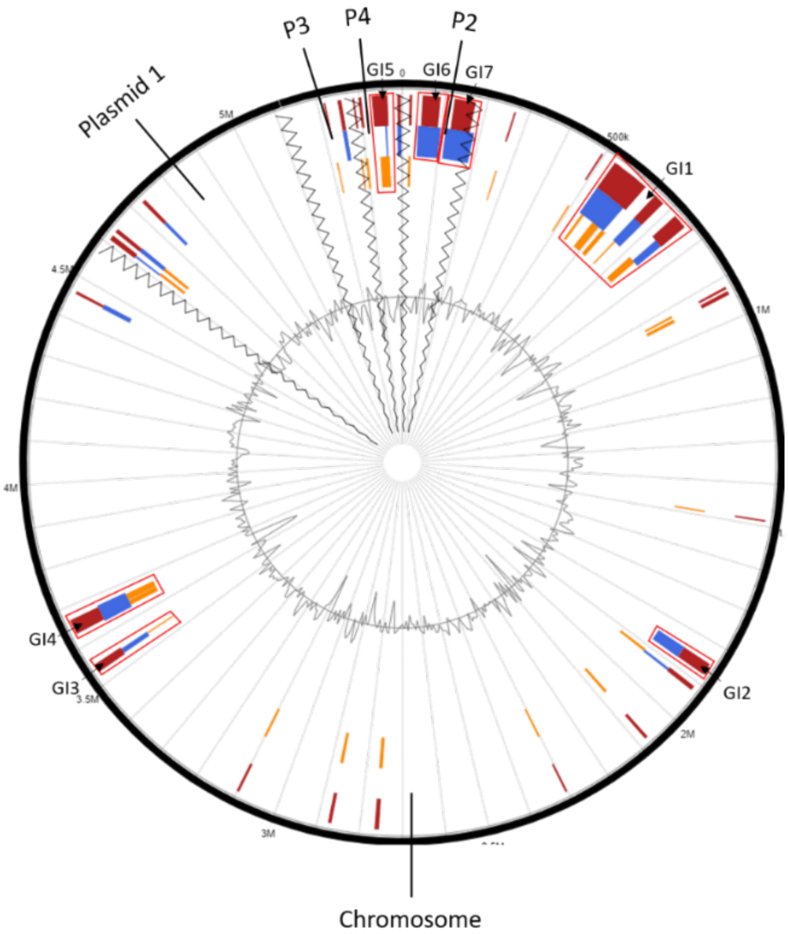

Figure S2: MAUVE alignment in progressive mode of *Pantoea agglomerans* DAPP-PG 734 plasmid 1 and *Pantoea vagans* C9-1 pPag3. Gene clusters for metabolic purpose and different features are indicated.

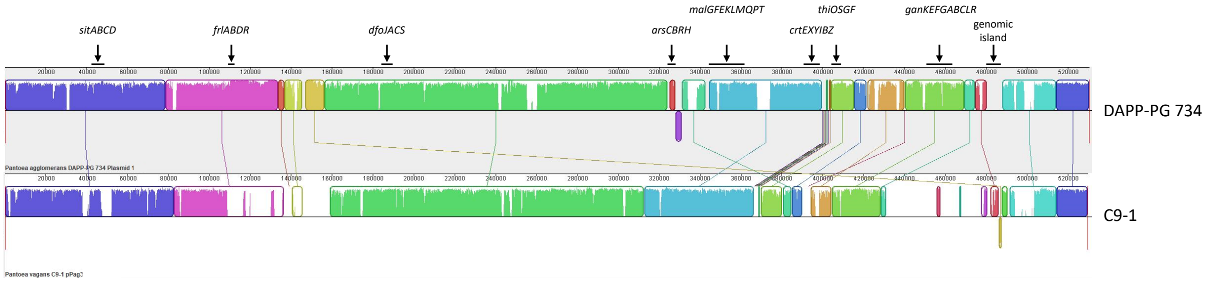

Figure S3: MAUVE alignment of *Pantoea agglomerans* DAPP-PG 734 plasmid 3 and *Pantoea vagans* C9-1 pPag1. Gene cluster for metabolic purpose and different features are indicated.

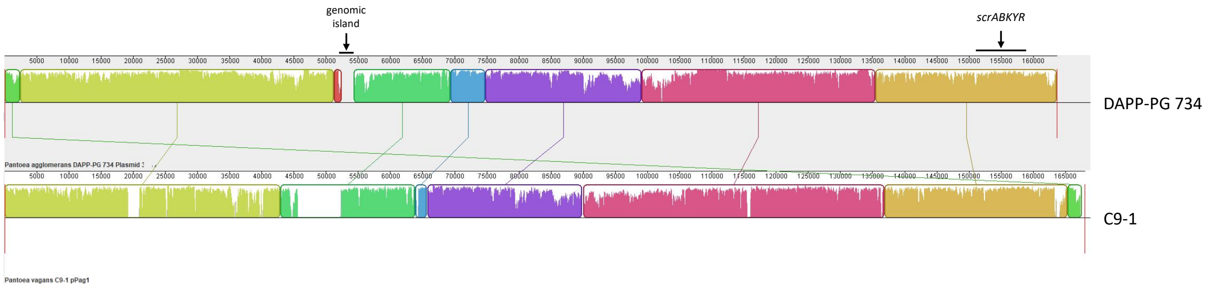

Figure S4: Gene cluster for degradation of fructoselysine in five strains of *Pantoea agglomerans*. Identical gene clusters within the genomes are shaded in grey and homologous genes are marked in the same color. The *frlABDR* gene cluster is colored in red and the additional subunit is colored in orange. White arrows represent no similarity to genes of other strains.

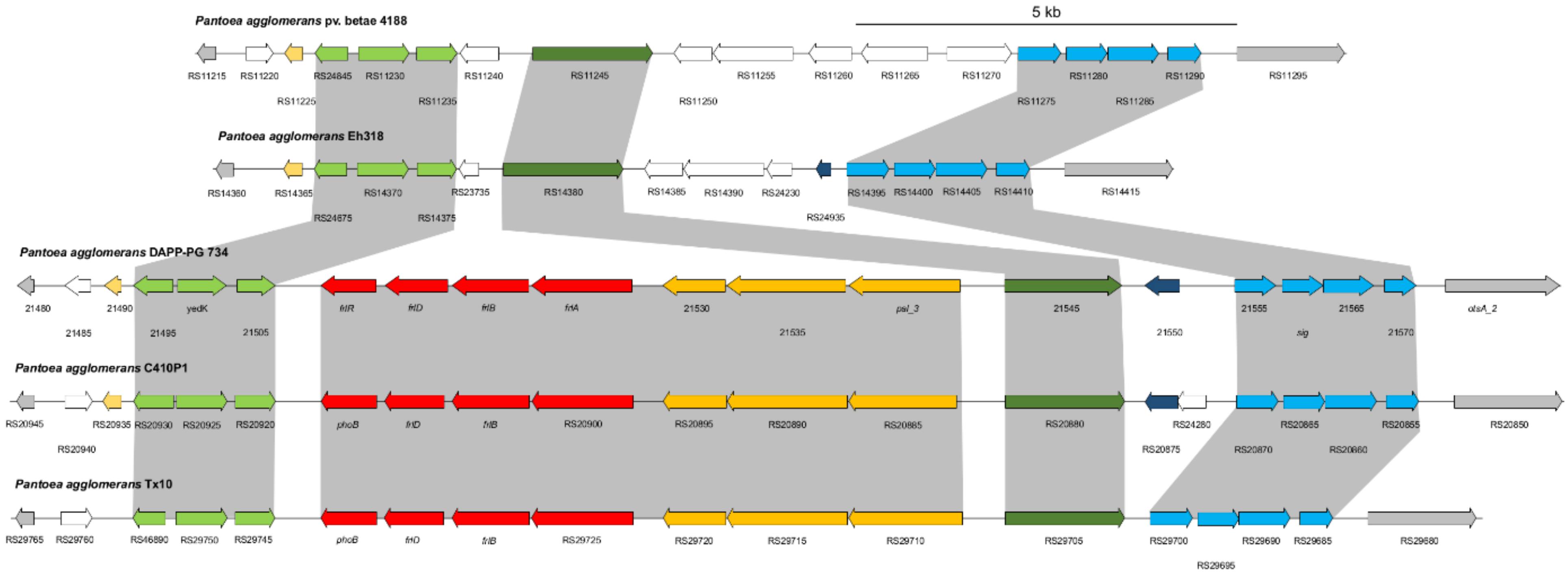

Figure S5: Gene cluster for biosynthesis of dapdiamide E in five *Pantoea* spp. The gene cluster for biosynthesis of dapdiamide E is shaded in grey, while the needed genes are colored in green. Conserved homologous genes, which are not part of the dapdiamide E cluster, are colored in yellow or in blue. White colored arrows represent no similarity to other genes. Pseudogenes are marked as dashed colored arrows.

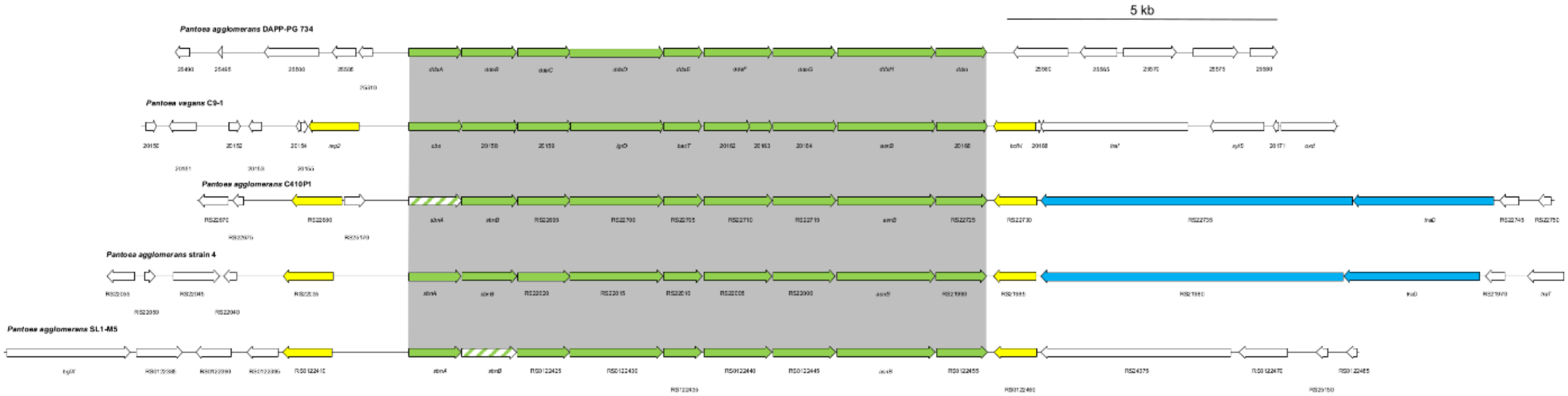

Figure S6: Gene cluster for biosynthesis of antibiotic B025670 in six *Pantoea* spp. The gene cluster for biosynthesis of antibiotic B025670 is shaded in grey, while the required genes are colored in violet. Homologous genes are marked in the same color. White colored arrows represent no similarity to other genes. A red asterisk represents a contig breaks within the sequenced genome.

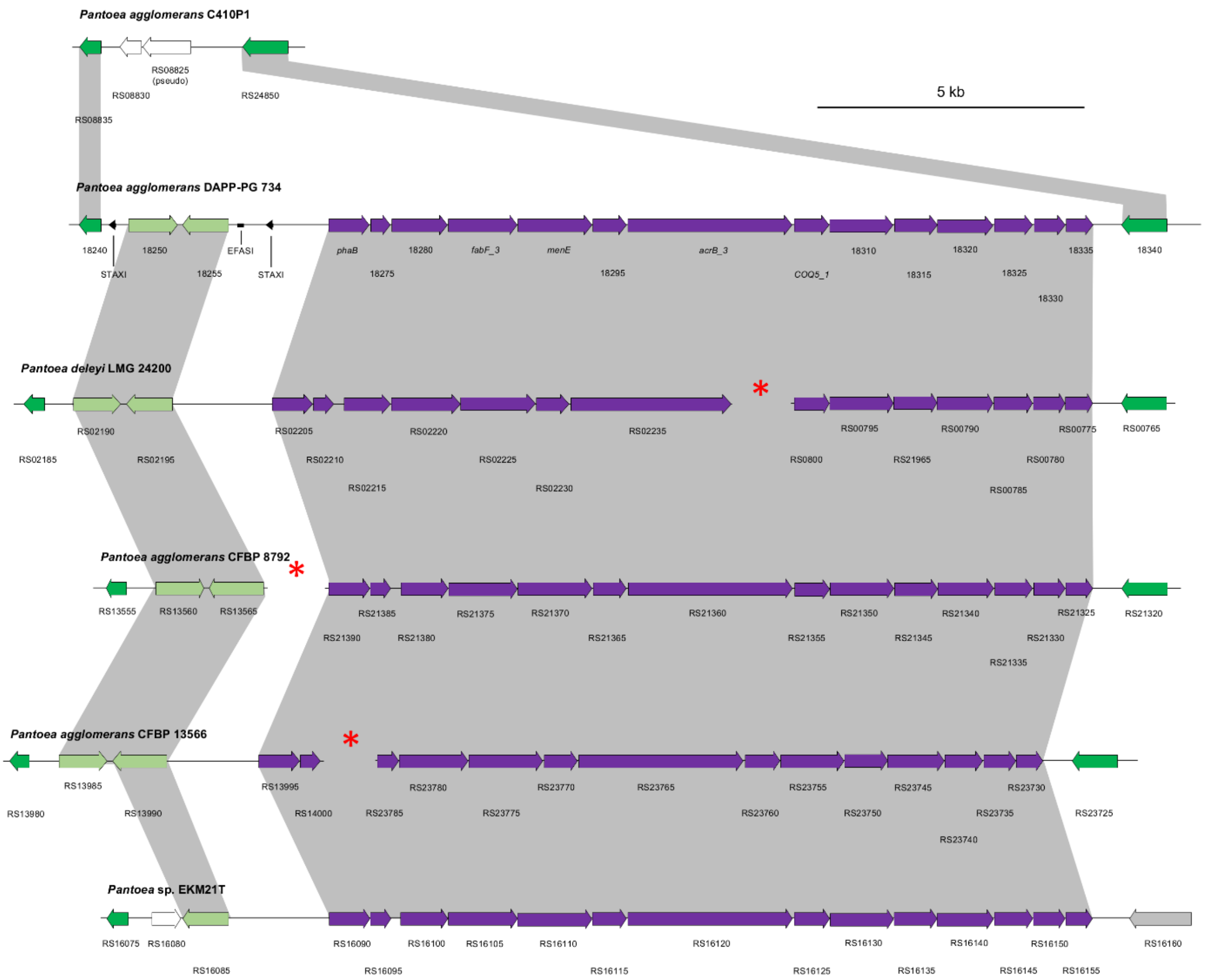

Figure S7: Gene cluster for type VI secretion system 1 (T6SS-1) in five *Pantoea* spp. The grey shadings show the conserved regions. Green marked arrows represent gene coding domains identified by Boyer et al. [17], and yellow painted arrows are not described genes by Boyer et al. [17] but are conserved genes among the *Pantoea* T6SS-1 loci [5]. The grey labelled arrows show the flanking sites of each strain. The red arrows stand for the *vgrG* and *hcp* genes. Blue- and light salmon-colored arrows represent homologous genes but are not part of the conserved region while the non-colored arrows (white) do not belong to the conserved genes and are not similar to other genes. Dashed colored arrows represent pseudogenes.

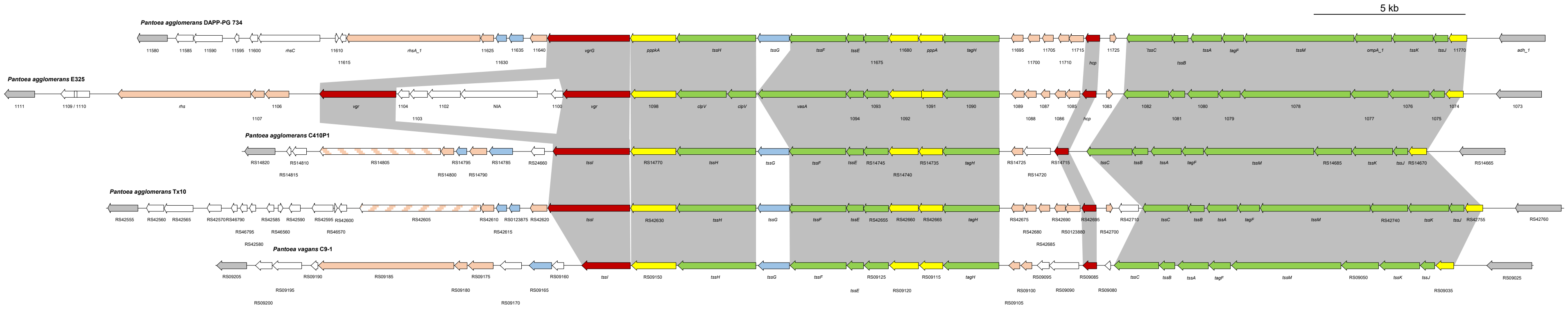

Figure S8: Gene cluster for type 6 secretion system 6 (T6SS-6) in five *Pantoea agglomerans*. This figure shows the genes involved for the biosynthesis of T6SS-6. The grey shading shows the identical cluster within the genome. Green marked arrows represent identical genes and grey labelled arrows shows the flanking sites of each strain. The red arrows identify the *vgrG* and *hcp* effector genes. Dashed colored arrows represent pseudogenes.

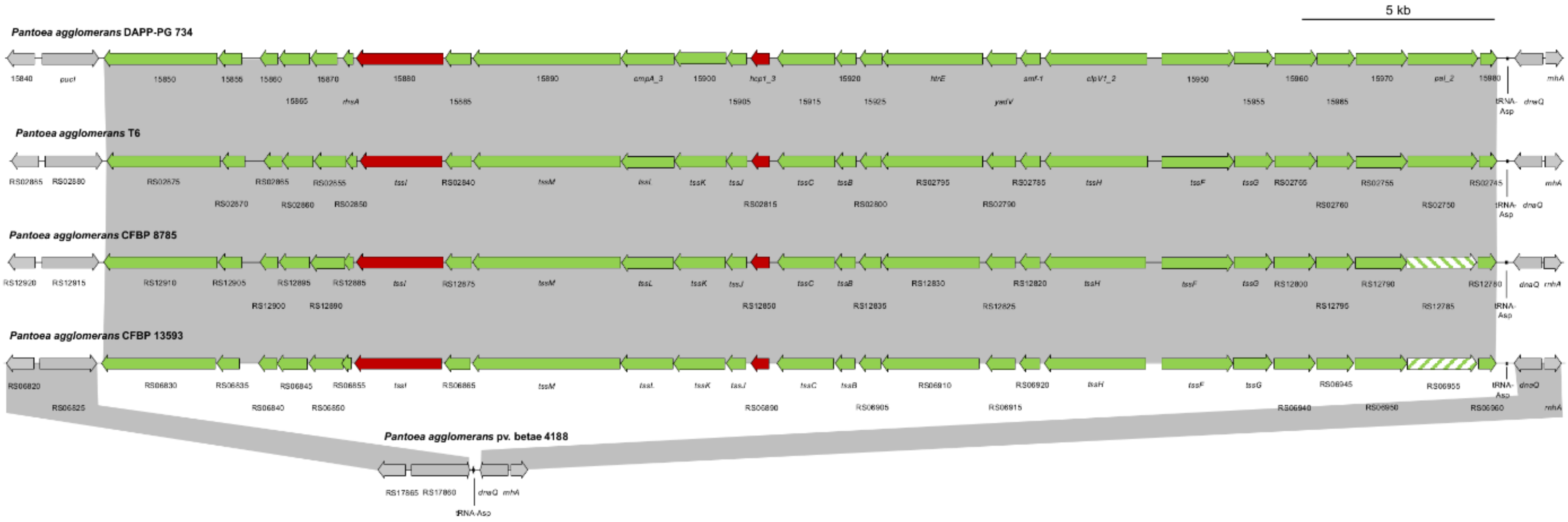

Figure S9: Gene cluster for biosynthesis of enterobactin in four *Pantoea* spp. The *ent-fep* gene cluster is shaded in grey, while the responsible genes are colored in green. Yellow colored arrows represent homologous genes but are not conserved genes. The flanking sequences are indicated by grey colored arrows.

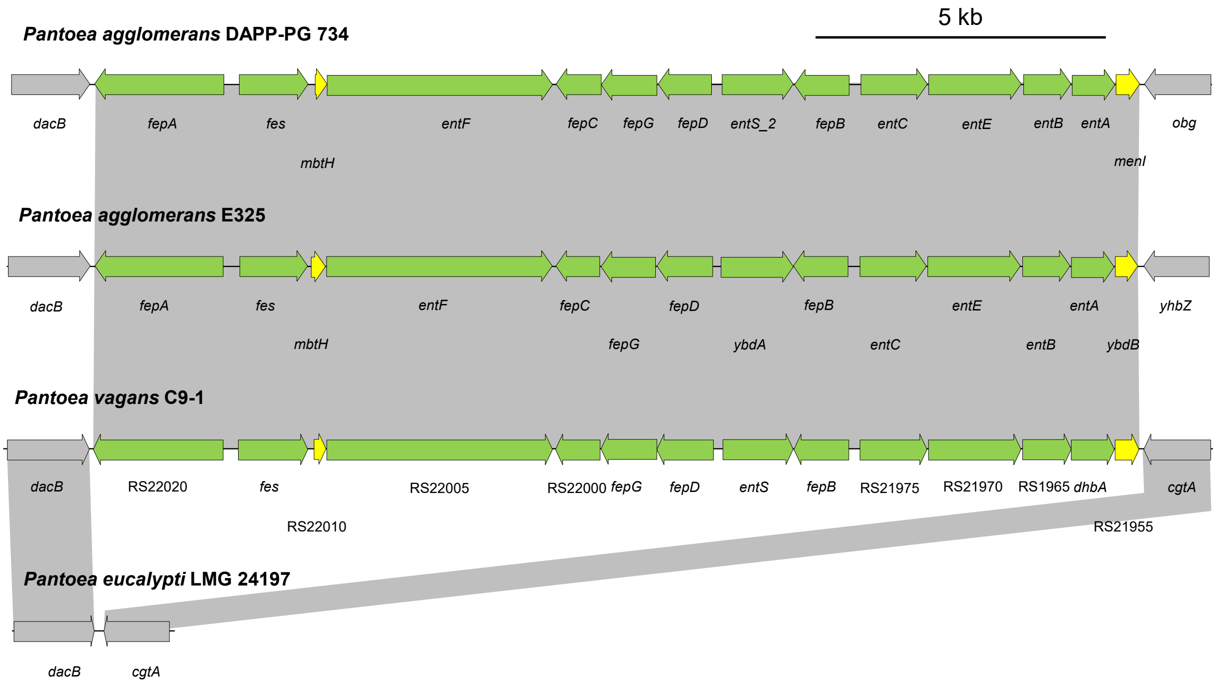

Figure S10: Gene cluster of the autoinducer biosynthesis *pagRI* in six *Pantoea* spp. The genes *pagRI* are colored in green and identical gene region within the genomes of related strains are shaded in grey. Blue and grey colored arrows represent homologous genes. Non-colored arrows (white) have no orthologs in the strains shown.

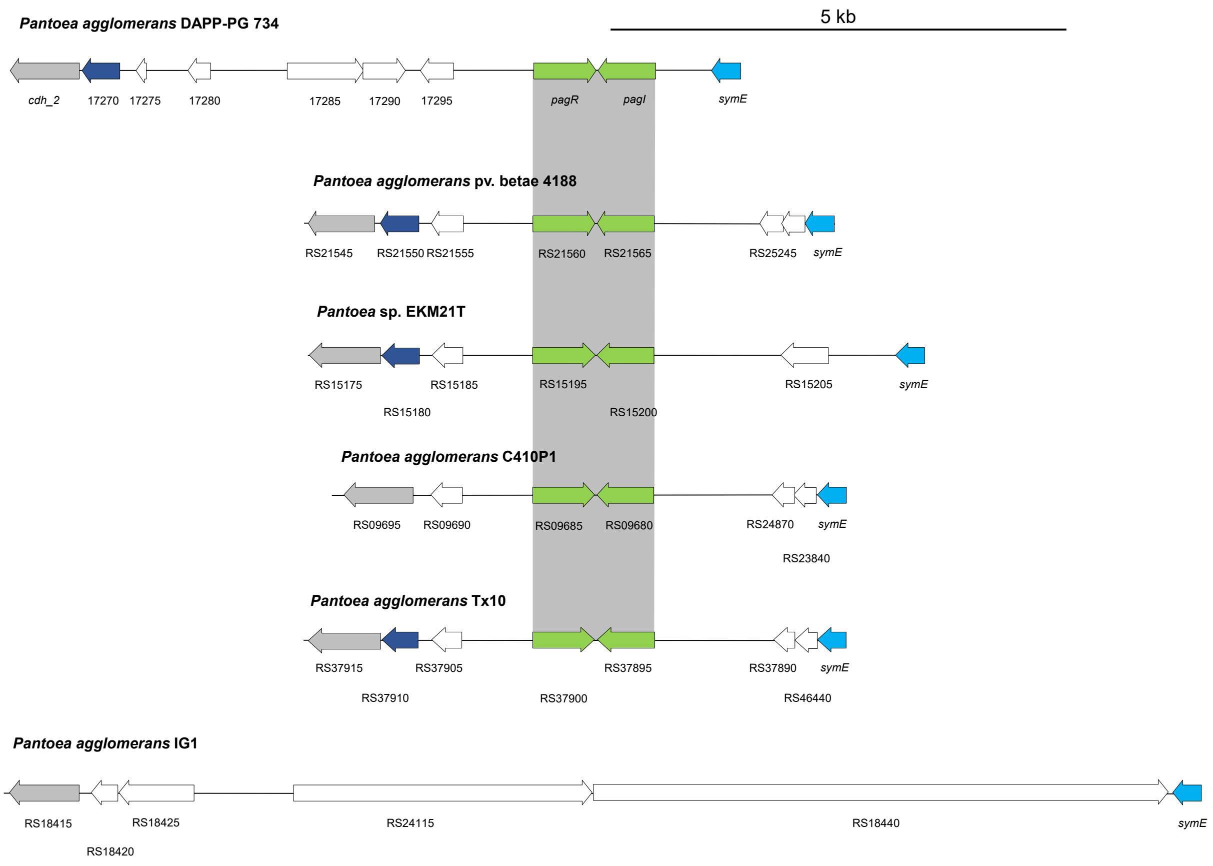

Figure S11: Gene cluster for biosynthesis of exopolysaccharide (EPS) in four *Pantoea agglomerans*. Identical genes within a genomic range across the compared strains are shaded in grey, while the responsible genes for the biosynthesis of EPS are colored in violet. Homologous genes, which are not part of the EPS cluster, are grouped by related colored arrows and pseudogenes are marked as dashed colored arrows. White arrows represent no similarity to genes in related strains.

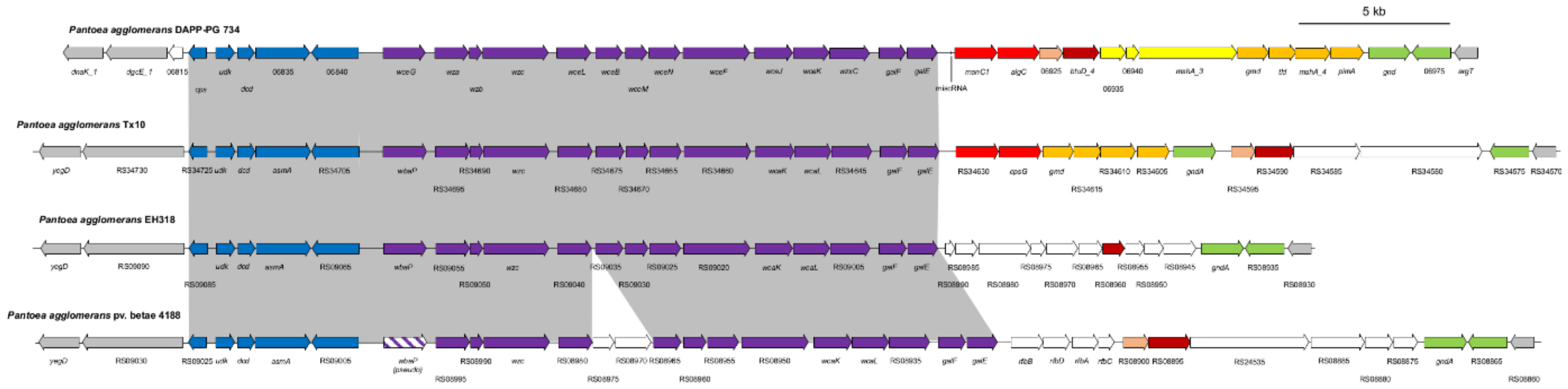

Supplemental references

1. Smits THM, Rezzonico F, Kamber T, Goesmann A, Ishimaru CA, Frey JE, Stockwell VO, Duffy B: Metabolic versatility and antibacterial metabolite biosynthesis are distinguishing genomic features of the fire blight antagonist *Pantoea vagans* C9-1. *PLoS One* 2011, 6(7):e22247.
2. Smits THM, Rezzonico F, Pelludat C, Goesmann A, Frey JE, Duffy B: Genomic and phenotypic characterization of a non-pigmented variant of *Pantoea vagans* biocontrol strain C9-1 lacking the 530 kb megaplasmid pPag3. *FEMS Microbiol Lett* 2010, 308(1):48-54.
3. Dippel R, Boos W: The maltodextrin system of *Escherichia coli*: metabolism and transport. *J Bacteriol* 2005, 187(24):8322-8331.
4. Delangle A, Prouvost A-F, Cogez V, Bohin J-P, Lacroix J-M, Hugouvieux Cotte-Pattat N: Characterization of the *Erwinia chrysanthemi gan* locus, involved in galactan catabolism. *J Bacteriol* 2007, 189(19):7053-7061.
5. De Maayer P, Venter SN, Kamber T, Duffy B, Coutinho TA, Smits THM: Comparative genomics of the type VI secretion systems of *Pantoea* and *Erwinia* species reveals the presence of putative effector islands that may be translocated by the VgrG and Hcp proteins. *BMC Genomics* 2011, 12:576.
6. Moretti C, Rezzonico F, Orfei B, Cortese C, van den Burg HA, Onofri A, Firrao G, Ramos C, Smits THM, Buonaurio R: Synergistic interaction between the type III secretion system of the endophytic bacterium *Pantoea agglomerans* DAPP-PG 734 and the virulence of the causal agent of olive knot *Pseudomonas savastanoi* pv. savastanoi DAPP-PG 722. *Mol Plant Pathol* 2021, 22(10):1209-1225.
7. Fukaya Y, Takemura M, Koyanagi T, Maoka T, Shindo K, Misawa N: Structural and functional analysis of the carotenoid biosynthesis genes of a *Pseudomonas* strain isolated from the excrement of Autumn Darter. *Biosci Biotechnol Biochem* 2017, 82(6):1043-1052.
8. Yuan X, McGhee GC, Slack SM, Sundin GW: A novel signaling pathway connects thiamine biosynthesis, bacterial respiration, and production of the exopolysaccharide amylovoran in *Erwinia amylovora*. *Mol Plant-Microbe Interact* 2021, 34(10):1193-1208.
9. Wang L, Wang J, Jing C: Comparative genomic analysis reveals organization, function and evolution of *ars* genes in *Pantoea* spp. *Front Microbiol* 2017, 8:471.
10. Klein JM, Loper JE, Stockwell VO: Influence of endogenous plasmids on phenotypes of *Pantoea vagans* strain C9-1 associated with epiphytic fitness. *J Plant Pathol* 2017, 99 (special issue):81-89.
11. Reid SJ, Abratt VR: Sucrose utilisation in bacteria: genetic organisation and regulation. *Appl Microbiol Biotechnol* 2005, 67:312-321.
12. Bogs J, Geider K: Molecular analysis of sucrose metabolism of *Erwinia amylovora* and influence on bacterial virulence. *J Bacteriol* 2000, 182(19):5351-5358.
13. Rowley G, Hensen D, Felgate H, Arkenberg A, Appia-Ayme C, Prior K, Harrington C, Field SJ, Butt JN, Baggs E *et al*: Resolving the contributions of the membrane-bound and periplasmic nitrate reductase systems to nitric oxide and nitrous oxide production in *Salmonella enterica* serovar Typhimurium. *Biochem J* 2012, 441:755-762.
14. Gardner AM, Helmick RA, Gardner PR: Flavorubredoxin, an inducible catalyst for nitric oxide reduction and detoxification in *Escherichia coli*. *J Biol Chem* 2002, 277(10):8172-8177.
15. McArthur AG, Waglechner N, Nizam F, Yan A, Azad MA, Baylay AJ, Bhullar K, Canova MJ, De Pascale G, Ejim L *et al*: The Comprehensive Antibiotic Resistance Database. *Antimicrob Agents Chemother* 2013, 57(7):3348-3357.
16. Bertelli C, Laird MR, Williams KP, Simon Fraser University Research Computing Group, Lau BY, Hoad G, Winsor GL, Brinkman FSL: IslandViewer 4: expanded prediction of genomic islands for larger-scale datasets. *Nucleic Acids Res* 2017, 45(W1):W30-W35.
17. Boyer F, Fichant G, Berthod J, Vandenbrouck Y, Attree I: Dissecting the bacterial type VI secretion system by a genome wide in silico analysis: what can be learnt from available microbial genomic resources? *BMC Genomics* 2009, 10:104.
